# A Modern Collaborative Behavior Analytic Approach to Incidental Naming

**DOI:** 10.1007/s40614-024-00399-0

**Published:** 2024-02-21

**Authors:** Amanda Gilmore, Dermot Barnes-Holmes, Maithri Sivaraman

**Affiliations:** 1 School of Psychology, Ulster University, Northern Ireland, UK; 2 Department of Health and Behavior Studies, Teachers College, Columbia University, New York, NY USA

**Keywords:** Incidental bidirectional naming, Relational frame theory, Verbal behavior development, Contextual cues

## Abstract

An important distinction has been drawn within the behavior-analytic literature between two types of naming. Naming that is reinforced is referred to as bidirectional naming, and naming that is not reinforced is referred to as incidental bidirectional naming. According to verbal behavior development theory children who demonstrate incidental naming have developed a verbal behavioral cusp, and often learn new language more rapidly as a result. A growing body of research has assessed incidental naming using what is described as an incidental naming experience, in which novel stimuli are presented and named by a researcher but with no direct differential reinforcement for subsequent naming responses by the participant. According to relational frame theory, such studies on incidental naming have typically involved presenting contextual cues that likely serve to establish the name relations between an object and its name. As such, contextual cues may play a critical role in the emergence of incidental naming responses, but there are no published studies that have systematically tested the potential role of contextual cues in relation to incidental naming. The current article provides a narrative review of the incidental naming literature, highlighting variables that remain to be explored in future research.

## Introduction

B. F Skinner’s contribution to the field of behavior analysis is renowned, Skinner being distinguished as one of the most influential natural scientific experimental psychologists (Morris et al., 2005). Skinner (1978) noted in his book *Reflections on Behaviorism and Society* that his work on the subject *Verbal Behavior* (1957) would prove to be his most important contribution, proposing a range of verbal operants, including mands, echoics, tacts, and autoclitics in its analysis. As is well-known, Skinner’s work on verbal behavior was heavily criticized in a review by Noam Chomsky (1959), and some have argued that it marked the rise of cognitive psychology and the demise of behaviorism (Palmer, 2006). Although *Verbal Behavior* did not generate anywhere near the level of basic research as did Skinner’s earlier work largely with nonhuman animals (but see Lamarre & Holland, 1985; Lodhi & Greer, 1989), it was fundamental in generating applied behavior-analytic approaches to remediating language deficits in young children, particularly those with diagnoses associated with developmental delays (McLaughlin, 2010; Sundberg & Michael, 2001). Furthermore, the concepts contained in *Verbal Behavior* have continued to contribute to behavioral research. For example, Horne and Lowe (1996) provided an analysis of the phenomenon of stimulus equivalence (e.g., Sidman, 1994) in terms of naming, using some of Skinner’s verbal operants (e.g., echoics, tacts, and intraverbals). Others attempted to incorporate many of the concepts presented in *Verbal Behavior* into other behavioral theories of human language, including relational frame theory (RFT, Hayes et al., 2001) and verbal behavior development theory (VBDT, Greer & Ross, 2008; Greer & Speckman 2009; see also Sivaraman et al., 2023).

One research area in which the concepts of Skinner’s (1957) *Verbal Behavior* have had considerable and continuing impact is in the conceptual and empirical analyses of naming, which has emerged as a prominent focus within the behavior-analytic literature. As noted above, a rise in the interest of naming within behavior analysis emerged primarily from the work of Horne and Lowe (1996) and their colleagues. In particular, these researchers used their account of naming to explain why unreinforced or untrained responses emerged in matching-to-sample performances during tests for equivalence relations. In a stimulus equivalence experiment, a participant may be trained to match two arbitrary stimuli (e.g., A1–B1) and then, during a test for a symmetrical relation, the participant may reverse that matching response (e.g., B1–A1) in the absence of differential reinforcement, instruction, or programmed prompting. Horne and Lowe (1996) argued that the symmetrical response may emerge because a verbally able participant could name each of the stimuli repeatedly during the Match to Sample (MTS) training (i.e., “A1–B1–A1–B1–A1–B1”), thus generating a bidirectional relation between the two stimuli, which would be observed during the MTS symmetry test. Horne and Lowe developed other naming-based accounts of stimulus equivalence relations and categorizing behaviors (Horne et al., 2006) in general, but the important point here is that the focus on naming in their work was very much on using it to explain stimulus equivalence and emergent relational responding more generally.

**Fig. 1:**
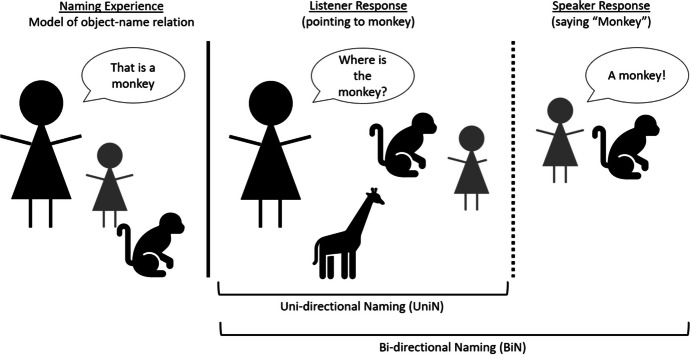
Incidental Uni- and Bidirectional Naming in a Child

Research on naming in behavior analysis has, however, extended well beyond its early focus on stimulus equivalence. Indeed, there has been growing interest in naming as a phenomenon in its own right and in particular its role in generating, or at least facilitating, more advanced language abilities (e.g., Miguel, 2018). In general terms, the concept of naming has been defined as the integration of listener and speaker behaviors within an individual through name–object and object–name interactions reinforced by social consequences (Olaff & Holth, 2020). Furthermore, naming researchers have distinguished between unidirectional naming (UniN) and bidirectional naming (BiN). The former occurs when an individual is able to identify a named object (by pointing at it) but fails to speak the name when asked to do so. The latter occurs when an individual is able to identify a named object and also speak its name (see Figure 1). Finally, it has been argued that naming is critical for the emergence of a child’s language developmental trajectory, with a consensus view that a verbal “vocabulary explosion” occurs around 18–24 months of age (Ganger & Brent, 2004; McMurray, 2007; Woodward et al., 1994).

It is critical to note that in the context of the current article, a potentially important distinction has been drawn between two types of naming, which seems to be directly relevant to a child’s vocabulary explosion. In particular, researchers have distinguished between naming that appears to require direct instruction or reinforcement versus naming that does not, the latter being referred to as incidental naming (Gilic & Greer, 2011; Olaff & Holth, 2020). That is, a child with incidental naming simply observes an individual stating a name in the presence of a novel object, which has been referred to as the “naming experience,” and subsequently the child responds correctly as a listener and a speaker. Of course, this distinction may be important in terms of developing a better behavior-analytic understanding of the variables that are involved in promoting or generating the critical vocabulary explosion that characterizes language development in neurotypical children. In the first part of the current article, we will focus on the distinction between naming and incidental naming, considering both conceptual and empirical analyses in the literature. In the second part of the article, we will examine incidental naming in more detail and draw on recent work in VBDT and RFT (see Sivaraman et al., 2023) to suggest how research on incidental naming may be pursued in future years.

## The Concept of Naming and its Subcomponents

The discipline of behavior analysis employs a wide range of technical terms and related acronyms that are customarily used throughout the literature. To nonexperts, the terms may be confusing, particularly when specific words are commonly used in the field, but which have less precise definitions in general language (e.g., the concept of chaining; Cooper et al., 2020). Of course, the term “naming” is widely used in everyday language, but it has acquired a more technical definition in behavior analysis, based initially on the seminal work of Horne and Lowe (1996). The authors argued that naming may be usefully considered a higher-order operant that involves the amalgamation of conventional listener and speaker components within an individual to form a bidirectional relation in a child’s behavioral repertoire. In particular, listener, echoic, and tact behaviors are seen as combining into a higher-order naming operant, such that reinforcement of a listener response may produce a speaker response or vice-versa, in the absence of additional training or instruction. Horne and Lowe’s naming theory thus constitutes an extension of Skinner’s *Verbal Behavior* (1957), which first defined echoics and tacts as verbal operants.

Almost 10 years after Horne and Lowe’s early work, research on naming began to focus on how naming could be instrumental for children to learn new language incidentally. For example, Greer et al. (2005) reported a study in which they used multiple exemplar instruction to establish incidental naming. They identified three children all of whom could respond correctly to listener trials during incidental naming trials but did not respond correctly to speaker trials prior to the experiment. During the study, researchers trained listener and tact responses across a number of exemplars, and once these behaviors had been established, they tested the children on their listener and speaker responses with a novel set of stimuli. All three children showed clear evidence of speaker incidental naming with the novel stimuli without further reinforcement or instruction; listener incidental naming was already present therefore incidental bidirectional naming (Inc-BiN) was established. Prior to the intervention none of the children had shown this form of incidental naming. Based on this and subsequent research, Greer et al. constructed verbal behavior developmental theory (VBDT), which sees naming as a critical progressive milestone in a child’s verbal development. As such, naming is defined as a verbal developmental cusp that facilitates children to acquire language faster and in new ways that they could not before the onset of the cusp (Greer & Du, 2015; Gilic & Greer, 2011; Sivaraman et al., 2021).

According to VBDT, children often learn new language more rapidly without direct instruction as a result of acquiring the naming cusp. During this developmental period a child’s naming repertoire may progress through joint attention and incidental exposure, which in the latter case involves simply observing a caregiver utter the name of an object or event in the environment in the absence of direct reinforcement or instruction. An increasing number of studies, following on from Greer et al. (2005), focused on such incidental naming abilities, and in particular attempted to develop interventions to produce such naming when it was found to be absent in a child’s repertoire. In particular, these studies typically tested the listener or speaker responses following a naming experience with an object without providing any differential consequences to generate incidental learning. If incidental naming did not occur, multiple exemplar instruction (MEI) or intensive tact instruction (ITI) were two commonly implemented interventions that have been used to induce Inc-BiN (e.g., Greer & Longano, 2010; Greer & Speckman, 2009; Olaff et al., 2017; Pérez-González et al., 2014).

Although research on incidental naming attracted increasing attention in the research literature, the concept of naming itself appeared to require greater precision. For example, Miguel (2016) argued that the use of the generic term “naming” may be misunderstood by both nonbehavioral and behavioral researchers. As a result, Miguel proposed the concept of “common bidirectional naming” (C-BiN) to distinguish it from other naming terms.^1^ The author argued that adding the identifier *bidirectional* would serve to emphasize the higher order operant of bidirectional relations in bidirectional naming (BiN). BiN being comprised of two parts: the unidirectional listener half of naming (UniN), and the speaker half of naming. UniN refers to a child hearing the name of an object in the environment (e.g., “dog”) in the presence of the object; to evoke listener behavior, an example could include a caregiver saying “Look, that is a dog,” thus drawing the child’s attention to the dog and then asking the child immediately, or at a later point in time, “Where is the dog?” If the child orients toward the dog, or points at the dog, then a successful listener response has been established (Sivaraman et al., 2021). For BiN to emerge, the functions of speaker responses to the object itself need to occur (Olaff et al., 2017). Speaker naming thus seems to require an echoic repertoire, which involves a child successfully repeating words uttered by a caregiver (Greer & Longano, 2010). Once an echoic repertoire is established, it may allow for listener behavior to facilitate speaker behavior (Horne & Lowe, 1996). In effect, when a child hears a caregiver name an object, the child may subsequently orient towards the object upon hearing the name, when asked where is the object, and the child may also echo the name when asked “What is this?” Miguel (2016) argued that naming includes all speaker relations of listening, echoic, and speaker verbal operants that are acquired separately but combine to enable comprehension. The critical distinction here, however, is between the two naming behaviors (listener and speaker) comprising BiN.

**Fig. 2:**
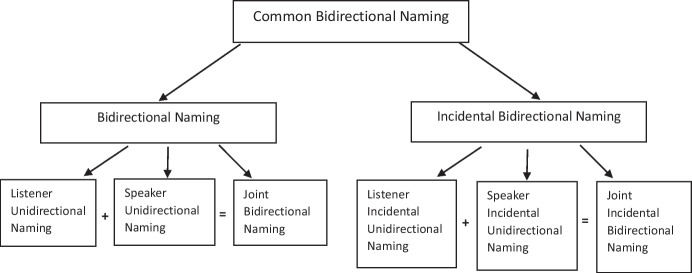
A Schematic Representation of the Proposed Classification of Common Bidirectional Naming (Hawkins et al., 2018)

In more recent years, the technical nomenclature of naming has been further refined in an effort to systematize the distinction between listener and speaker naming and the concept of incidental naming. Hawkins et al. (2018) deconstructed part of Miguel’s (2016) naming framework into a technical classification with six different subtypes of common bidirectional naming (see Fig. 2). As well as drawing from primary research in bidirectional naming (listener and speaker behavior), which tested for the emergence of speaker behavior when listener behavior was trained or vice-versa, the proposed taxonomy drew from research on incidental naming. In particular, Hawkins et al. acknowledged that Greer and Ross (2008) described “full naming” as the acquisition of novel speaker and listener behavior via an incidental naming experience (i.e., without direct teaching). As such, the authors argued that it may be useful to distinguish between bidirectional naming (BiN) and incidental bidirectional naming (Inc-BiN). Furthermore, each of the two distinct categorizations were organized into three subtypes that are seen as the prerequisites for the composite category of naming behavior in toto (Hawkins et al., 2018).

The subtypes within bidirectional naming are congruent with the subtypes within incidental naming. For instance, bidirectional naming consists of listener unidirectional naming plus speaker unidirectional to form joint bidirectional naming. Likewise, incidental naming consists of listener incidental unidirectional naming plus speaker incidental unidirectional naming to form joint incidental bidirectional naming. VBDT researchers posit that the distinction among the terms is perspicuously found in the procedures used to assess the different variations of naming (Kleinert-Ventresca et al., 2023). In a typical BiN procedure, researchers test the emergence of untaught listener/speaker behavior following the teaching of speaker/listener behavior, respectively. In other words, one topography is trained (listener or speaker), and the other topography emerges without training. Testing for listener responses usually involves a researcher presenting an array of stimuli with the instruction “Point to *object*” or “Where is *object name*?” Whereas, testing for speaker responses normally involves a participant being asked ‘What is this?’ (Hotchkiss & Fienup, 2020; Greer et al., 2005; Fiorile & Greer, 2007). Hawkins et al. (2018) argued that a complete test for bidirectional naming involves testing speaker naming following listener training, and testing listener naming following speaker training, within the same individual.

In the naming taxonomy proposed by Hawkins et al. (2018), the first three subtypes (1) listener naming; (2) speaker naming; and (3) joint bidirectional naming involve direct training or instruction for one of the response topographies (i.e., listener or speaker). For example, to demonstrate the third subtype, a child might be directly trained or instructed to identify a stimulus from an array (listener naming) and then tested without further instruction for speaker naming (or vice-versa, train speaker naming and test listener naming). The remaining three subtypes comprise incidental naming and map onto the first three subtypes but without any direct training or instruction for either of the response topographies. In particular, the incidental naming subtypes only involve a naming experience in which a child simply observes an object and listens to its name being uttered. Subtype 4, for example, involves the emergence of untaught listener behavior following a naming experience. In particular, listener incidental unidirectional naming emerges (i.e., Inc-UniN) when individuals emit untaught listener behavior (e.g., pointing) but not speaker behavior after hearing the tact of an object without direct teaching. In contrast, Subtype 5 refers to speaker incidental unidirectional naming, which involves an individual tacting an object without having been directly trained or instructed in listener or speaker responses for that object. Finally, Subtype 6 deals with incidental bidirectional naming (Inc-BiN) in which both listener and speaker responses emerge without direct training or programmed reinforcement following a naming experience.

In the assessment of Inc-BiN, Hawkins et al. (2018) drew from previous research that employed a match-to-sample (MTS) procedure, which allowed researchers to test the names for novel stimuli without direct or programmed instruction for either speaker or listener responses (Greer et al., 2007). The procedure initially requires the participant to match a stimulus with other stimuli, as instructed by the vocal antecedent “Match *object name*,” which is delivered by the researcher (i.e., the naming experience; Kleinert-Ventresca et al., 2023). In this case, the participant is exposed to the name of an object but without any differential reinforcement for engaging in either listener or speaker behaviors (i.e., the participant is simply required to match the object with a similar object within the MTS procedure). However, the participant may be subsequently tested for Inc-UniN and/or Inc-BiN. For example, the participant might be asked to point to the object upon hearing its name, and to tact the object without any request to do so (pure tact) or to tact the object when asked “What is this?” (impure tact) while the researcher points at the object.

Hawkins et al. (2018) suggested the proposed classification of naming into six subtypes may enable researchers to compare naming studies more systematically. Doing so, it was argued, may enhance the precision of the technical language that is employed in the study of naming within the behavior-analytic literature. The extent to which the wider literature will adopt to the taxonomy remains to be seen, but recent research (Yoon et al., 2023) has begun to draw on the work of Hawkins et al.

## Relational Frame Theory on Naming

The previous discussion has highlighted how naming research has evolved from the study of stimulus equivalence to the concept of verbal developmental cusps, and the acquisition of incidental naming from the perspective of VBDT. As indicated previously, we will also consider naming from the perspective of RFT (see Sivaraman et al., 2023), another modern behavior-analytic theory that has focused on the study of human language. In one sense, RFT is an extension of Skinner’s (1957) text, *Verbal Behavior,* but drew heavily on the phenomenon of stimulus equivalence. Skinner’s concept of verbal behavior is based largely on a direct contingency-based account, which defines verbal behavior as being reinforced through the mediation of another organism that has been conditioned to provide that reinforcement (Hayes et al., 2001; Barnes-Holmes et al., 2000). RFT is clearly anchored in the principles of behavior analysis, in that it draws on the concepts of operant and respondent conditioning. According to RFT, language involves learning to relate stimuli, such as words and objects, in an arbitrarily applicable manner (i.e., not based solely on their physical or formal properties). Various patterns of such relational responding, referred to as relational frames, are established via a history of operant conditioning, across multiple exemplars, sometimes defined as multiple exemplar training (MET; Barnes-Holmes & Barnes-Holmes, 2000). In terms of RFT, MET is a generic concept that refers to any multiple exemplar training that serves to generate a particular pattern or patterns of arbitrarily applicable relational responding (AARR). Given that RFT is an operant account, the definition of MET is not constrained by the topographies of the stimuli or responses involved in the training. As such, MET may be seen as a broad umbrella term that covers more specific concepts such as multiple exemplar instruction (MEI; e.g., Greer et al., 2007; Greer & Speckman, 2009), multiple response-exemplar training (MRET; Olaff et al., 2017), or mixed operant instruction (MOI; see Cooper et al., 2020).

The process of AARR is established for a child in its early language interactions with the wider verbal community, and gradually increasingly complex patterns of AARR are generated (e.g., from listener naming, to speaker naming, to rule-governed behaviors, and analogical reasoning). One of the critical defining properties of relational frames, or AARR, is that increasingly complex patterns of verbal behavior (i.e., relating) may occur without direct training or reinforcement (Barnes-Holmes & Harte, 2022). In particular, the extended history of AARR serves to establish specific contextual cues, which control the relational responding in a manner that extends beyond the formal or physical properties of the related stimuli (Stewart, 2018). Consider, for example, reinforcing an object–name relation in one direction and testing for the reversed symmetrical, or *mutually entailed,* relation in the absence of reinforcement (i.e., the tested relation is derived from the reinforced relation). In concrete terms, a child might be shown an object and told its name (object–name relation) and subsequently asked to identify the named object (name–object relation). For the name relation to be defined as a derived and mutually entailed relation, the child must identify the object (upon hearing its name) without explicit reinforcement or further training. If explicit training is required, then the naming response cannot be defined as derived (because both object–name and name–object relations were explicitly taught; Barnes-Holmes et al., 2018). According to RFT, a child may learn to produce derived naming based on an operant history of multiple exemplars, sometimes referred to as multiple-exemplar training or MET. In particular, MET reinforces object–name and name–object relational responses across a number of stimulus exemplars, and then tests for derived naming (mutual entailment) using novel names and objects (not used during the training; e.g., Luciano et al., 2007). The core postulate is that specific contextual cues for derived naming are reinforced across MET, and thus eventually these cues may control such naming in the absence of direct training (i.e., contextual control generalizes to novel names and objects).

As noted above, RFT argues that increasingly complex patterns of relational responding may be generated via a history of MET. For example, relational responding may be characterized by the properties of mutual entailment, combinatorial entailment and transformation of functions (Gibbs et al., 2023). Mutual entailment refers to a derived bidirectional relation between two stimuli in a specific context, where responding in one direction leads to a relation in another direction within the same context. For example, if stimulus A is same as stimulus B, then the derived relation would entail that stimulus B is the same as stimulus A. Combinatorial entailment refers to the emergence of derived relations when two stimulus relations are combined. For example, if stimulus A is the same as stimulus B, and stimulus B is the same as stimulus C, then derived relations would be stimulus A is the same stimulus C, and stimulus C is the same stimulus A, in that given context, without any additional instruction or training. The transformation of functions refers to any change in the functional properties of a stimulus based on the derived relations it has with other stimuli. For example, if stimulus C is in a derived “sameness” relation with stimulus A, and C is established as highly appetitive through direct stimulus pairing (e.g., respondent conditioning), then stimulus A may also acquire appetitive functions without any explicit pairing or conditioning (Barnes-Holmes et al., 2001). The properties of mutual entailment, combinatorial entailment and the transformation of functions play an important role in distinguishing between listener and speaker naming.

To illustrate, consider that AARR always involves a transformation of functions in accordance with an entailed relation or relations. In the case of UniN, it has been argued that the relevant transformation of functions is relatively limited. That is, a child need only orient toward a novel object (or point toward it/pick it up) when a caregiver names that object. For BiN, however, the child not only orients to the object but also vocalizes the word that was heard when the caregiver named the object. Some researchers have argued that the additional transformation of functions involved in BiN (speaking as well as orienting), relative to UniN, renders the former a basic relational frame (involving combinatorial entailment; see Greer et al., 2005; Luciano et al., 2007), whereas the latter seems only to be characterized by mutual entailment (see Sivaraman et al., 2023).

In distinguishing between UniN and BiN, RFT also focuses on the controlling functions of specific contextual cues. These cues, such as linguistic request terms (e.g., “Where is *object*–*name*?” or “Look, that is an *object*–*name*”), and/or paralinguistic gestures such as pointing to or looking at the object are discriminative for a child to orient towards (or point or reach for) the object. This type of interaction between a child and caregiver may be repeated with various objects in numerous settings with different individuals, but the contextual cues (linguistic or paralinguistic) provided from the social community remain relatively precise and consistent. From an RFT perspective, this type of learning may be interpreted as MET, but occurring in a relatively unprogrammed way in the natural environment rather than a classroom or research setting (Sivaraman et al., 2023).

According to RFT, the contextual cues (linguistic and paralinguistic) may serve as stimuli that establish the arbitrary relation between a word and an object, and also control a specific response to that word or object; the former cue is referred to as a Crel (i.e., the context for the relation) and the latter as a Cfunc (the context for the response function) (Törneke, 2010). Consider, for example, a naming episode between a parent and a child on a trip to the zoo, with a parent who says “Look, it’s an aardvark” upon seeing an example of the animal. In this case, the phrase “it’s a” may function as a Crel for establishing a mutually entailed relation between the animal and the sound “aardvark,” and the word “Look” may function as a Cfunc for actually gazing at the animal. Additional actions by the parent, such as pointing to the aardvark and encouraging shared engagement (crouching down beside the child while looking at the animal) may serve as additional Crel and Cfunc cues. If the child has a relatively limited reinforcement history with such cues, the child may not readily learn the name of the animal, without additional prompting and reinforcement. For example, the child may fail to point at the aardvark when asked to do so by the parent. If this occurs, the parent may again point to the animal and name it, saying, for example, “It’s an aardvark, don’t you remember, he’s funny looking isn’t he?” If, however, the child has an extensive reinforcement history with the relevant Crel and Cfunc cues, the child may identify the aardvark correctly when asked to do so, following only a single naming episode.

It has been argued that the transformations of stimulus functions involved in a UniN listener response are relatively limited, in that the child simply orients toward the object and may either point to, or pick up, the object that was named by the caregiver. In contrast, the transition from UniN to BiN appears to involve a relatively complex transformation of functions because the child not only orients towards an object (listener behavior) but vocalizes the corresponding name of that object (speaker behavior) (Sivaraman et al., 2023). As such, RFT argues that the speaker half of naming marks a shift from mutually entailed relational responding. That is, UniN simply involves a bidirectional (mutually entailed) relation between hearing and orienting, whereas BiN involves bidirectional relations among hearing, orienting, and speaking (a combinatorial entailment among the three elements). The shift from mutual to combinatorial entailment in BiN thus marks the establishment of a basic or simple relational frame that incorporates a derived transformation of functions that is more complex than the transformation involved in UniN. Indeed, others have argued that the vocal utterances by a child in the speaker component of BiN are significant because it establishes when a child has learned to tact objects with understanding (Miguel, 2016). From an RFT perspective, once a child has learned to respond in accordance with the relational frame of BiN, given appropriate contextual cues, the emergence of incidental naming becomes more likely, provided that the relevant cues are present during a naming episode. For instance, if the contextual control is relatively well-established and precise, as demonstrated across previous multiple exemplars of BiN, incidental naming may then occur. For example, when a child hears the phrase “That is a guitar,” without a direct history of reinforcement while oriented towards the instrument, the child may then point at the object (Inc-UniN) along with vocalizing the name of the object (Inc-BiN). In effect, the child acquires the name “guitar” incidentally based on contextual cues, such as being oriented towards the object and hearing the phrase “That is a. . . .”

### VBDT and RFT: Employing Both in the Analysis of Incidental Naming

As noted earlier, researchers in VDBT have argued that the acquisition of BiN in a child’s naming repertoire is a prerequisite for a child to be able to learn names of novel stimuli incidentally without direct teaching or reinforcement. Indeed, and again as noted earlier, the growing body of research associated with the concept of incidental bidirectional naming has contributed to a proposed taxonomy classification by Hawkins et al. (2018), in an effort to discern the subtypes of naming within the literature. Given that the preceding section on RFT focused on the role of contextual cues in a BiN context, it may be useful to consider the role of these cues in the acquisition of Inc-BiN. We will do this here, by drawing on experimental studies within the literature related to the subtypes of incidental naming proposed by Hawkins et al.

From a VBDT perspective, Inc-UniN or Inc-Bin may be assessed following an incidental naming experience, which generally involved in earlier studies an MTS procedure using a novel stimulus with no direct teaching or reinforcement from the researcher. The experimenter typically vocalizes the name of the visual stimulus in the presence of the stimulus; for example, “Match spatula” (see Gilic & Greer, 2011) or “Match horse with horse” (see Hawkins et al., 2009). In some respects, this naming experience simulates a naturalistic setting, whereby a child learns listener and speaker object–name relations through observation alone. Key differences may be identified across studies, such as the stimuli used, the number of stimuli employed, or testing for the emergent components of naming using a contrived stimulus (e, g., “Match Zog”). From an RFT perspective, such studies on incidental naming have typically involved presenting contextual cues that likely serve to establish the relevant entailed relations and transformations of functions. For instance, when researchers present the vocal instruction “Match *object-name,*” the word “match” most likely functions as a Crel. In addition, any gestures (e.g., handing the object to the child) that the experimenter might produce may also function as a relevant Crel for establishing an entailed relation between the object and the name. As such, contextual cues may be playing a critical role in the emergence of incidental naming responses.

Let us consider a more naturalistic example of a naming experience involving a caregiver and a toddler playing with a toy octopus. The caregiver might pick up and place the octopus in front of the child, point to it and say “That is an octopus” while looking back-and-forth between the octopus and the child. These stimuli (i.e., saying “that is,” pointing, holding the toy) are seen as the Crel that specify the relation between the object and its name. It is important to highlight that some of these cues are linguistic while others are not. In particular, the verbal statement “That is an octopus” is a linguistic cue whereas the pointing, holding the octopus, and orienting back-and-forth are paralinguistic cues (also called deictic gestures in developmental psychology; Iverson & Goldin-Meadow, 2005). In the case of a toddler beginning to learn their first words, it seems likely that the paralinguistic cues control responding until the linguistic cues (e.g., “That is”) acquire symbolic properties (see Morford & Goldin-Meadow, 1992, for a study on gesture comprehension in preverbal toddlers).

It can be argued that as the child grows older there may be instances in which linguistic cues come to entirely control responding. As an example, I might say, “Look” while showing a 6-year-old child a rambutan, a novel fruit during a visit to the market. At this time, I say nothing to the child about its name. Once we get back home, a few minutes later, I might tell the child “remember the thing I showed you at the market, that’s called a rambutan.” In such a naming experience, all the cues presented are linguistic—i.e., the object and name are not presented simultaneously, there are no gestures involved, and only the vocal statement *relates* the object with its name. If the child were to respond correctly as a listener (i.e., pointing to the rambutan on a subsequent market visit) and as a speaker (saying “rambutan” on seeing the fruit), such performance may be deemed more advanced or complex compared to the earlier example involving the toddler and the octopus. As such, the paralinguistic cues and temporal contiguity (between the object and its name) may facilitate critical experiences in the child’s behavioral history that precedes such advanced performance.

Sivaraman et al. (2021) conducted a study in which they measured toddlers’ correct listener (Inc-UniN) and speaker responses (Inc-BiN) following a naming experience. In particular, during the naming experience, they presented the object and name nonsimultaneously by showing the child a novel object and then hiding it under a white cloth, pointing to the region of the white cloth and saying its name. They found that 16–22-month-old toddlers did not emit correct listener or speaker responses when objects and names were presented in this nonsimultaneous manner. If the foregoing analysis is juxtaposed against this empirical finding, one could argue that Sivaraman et al. removed one of the paralinguistic cues in the naming experience that controlled the toddlers’ responding (i.e., holding the object up while its name was uttered). Following multiple exemplar listener training, all participants subsequently responded correctly as a listener when objects and names were presented nonsimultaneously. That is, one could argue that posttraining, the other cues presented during the naming experience (i.e., pointing to the region of the cloth, and saying “that is a. . . .”) came to control the toddlers’ naming responses.

Of course, the foregoing argument is largely interpretive. At the time of writing, we were not aware of any published studies that systematically tested the potential role of contextual cues in relation to either Inc-UniN or Inc-BiN. In a subsequent section of the current article, therefore, we will outline a number of studies that might be conducted to explore the potential role of contextual cues in incidental naming. The purpose of this exercise is to encourage researchers from different theoretical perspectives to focus their combined efforts on advancing the study of what is clearly and critically an important verbal developmental milestone or behavioral cusp; the point at which children can learn the names of novel objects and events in the absence of direct instruction, reinforcement or prompting.

## Procedures to Present a Naming Experience

Before considering some potential directions for future research on incidental naming, it seems important to highlight a critical aspect of the methods used to test incidental naming, i.e., the naming experience. As such, simply based on the exposure to a novel object and its name during a naming experience, children with Inc-BiN respond correctly as a listener and as a speaker. Therefore, the naming experience is crucial to our understanding of incidental naming, and to designing robust experiments that facilitate the emergence of this behavior. Two categories of procedures to present a naming experience may be drawn from the literature since the inception of incidental naming studies, which we will label as (1) MTS procedure; and (2) stimulus pairing procedure with or without delayed probes. In the next section, we will offer a brief overview of these procedures (see Sivaraman & Barnes-Holmes, 2023, for a detailed overview of all empirical studies conducted using each of these methods.^2^

### MTS Procedure

As noted in the previous section, earlier incidental naming studies often involved an MTS task procedure in which the child was required to match a picture with an identical picture while the researcher provided an instruction “Match [name] with [name].” For instance, Gilic and Greer (2011) used sets of 3-D stimuli and each MTS trial began once the researcher had established joint attention with the child (i.e., the researcher affirmed that the child was looking at the novel object before delivering the instruction). The researcher then delivered specific instructions to match the sample with an identical stimulus in the comparison array (e.g., “Match spatula with spatula”). The authors stated that the elements of this procedure created an opportunity for incidental naming because the child was presented with a novel picture and heard its name. Several other studies have used this procedure as a means to present a naming experience (e.g., Cao & Greer, 2018; Greer et al., 2005; Hotchkiss & Fienup, 2020). All these studies subsequently tested participants on their listener and speaker responses to the novel stimuli presented during the MTS trials.

Two procedural details reported across these studies warrant additional consideration. First, in all of these studies some form of reinforcement was delivered for correct matching responses. For example, Cao and Greer (2018) and Gilic and Greer (2011) reported delivering social praise for correct matching responses, and Longano and Greer (2015) reported using either praise or edible reinforcers. It seems reasonable to surmise that delivering reinforcement for correct matching responses may be needed to maintain the participants’ motivation and may also mimic some naturalistic matching experiences that might involve praise from the caregiver. It is important to note that the other procedures reported in the literature to present a naming experience (detailed above) do not involve the delivery of programmed reinforcement following exposure to an object and its name. Second, a few studies reported conducting some MTS trials under no-reinforcement probe conditions with novel variations of the same stimulus (i.e., a novel type of spatula that was not previously reinforced). For instance, Gilic and Greer (2011) used one variant of a stimulus during MTS instruction whereas two variants were used during MTS probes, and no programmed reinforcement was provided for correct responses during the probes.

### Stimulus Pairing Procedure

The second approach to presenting a naming experience involves a stimulus pairing procedure, in which a researcher holds up the novel object/picture and simply states its name (e.g., Longano & Greer, 2015; Pérez-González et al., 2014). In particular, a visual stimulus is presented to participants either directly or on a computer screen, and the researcher points to the stimulus while simultaneously saying the name of the stimulus. These trials also involved the researcher affirming that joint attention had occurred (i.e., the participant looked at the visual stimulus while hearing the word). It is critical to note that no feedback or programmed consequences were provided for the participants’ observing responses. Similar to the studies that used MTS trials, researchers using stimulus pairing also conducted probes for listener and speaker responses following the naming experience session. Although some studies conducted these listener and speaker probes immediately after a naming experience session, others have reported delays from a few minutes to a few hours (e.g., Longano & Greer, 2015; Cao & Greer, 2018)

Kleinert-Ventresca et al. (2023) reported a procedural variation in the stimulus pairing naming experience. In particular, these researchers provided one stimulus pairing naming experience session as described above, and this was followed by a series of listener and speaker probes conducted from a few hours to a few days later. All of the participants in this study could emit correct listener responses but not correct speaker responses at enrolment (i.e., they had incidental unidirectional naming only). The authors hypothesized that the series of listener probes conducted on subsequent days served as an additional form of naming experience (i.e., in addition to the stimulus pairing) for the participants. This type of presentation has not been widely studied in the behavioral literature and its scope and utility across future research and practice remains to be seen.

### Variables that Remain to be Explored with Incidental Naming

Each of the category of studies described above provide evidence for Inc-BiN when the children demonstrate listener and speaker responses through object–name exposures alone; that is, in the absence of direct reinforcement. It has been argued that research on Inc-BiN is important because the ability to learn the names of stimuli in the absence of direct reinforcement is a critical behavioral cusp that facilitates the development of language skills in general (Greer et al., 2017). In studying what may be such an important behavioral “building block” it seems important to explore the key variables that may be involved in generating Inc-BiN. Doing so would not only provide important functional-analytic information concerning the behavioral process involved in Inc-BiN itself but could also be of use to practitioners who are seeking to facilitate Inc-BiN when it is found to be absent or relatively weak in a child’s behavioral repertoire. In this regard, we suggest three variables below that we suspect may be important to exploring Inc-BiN. We fully acknowledge that there are likely other variables involved but have focused on these three as a first step; furthermore, additional variables may well come to light during the course of experimental research in this area.

First, joint attention between the researcher and the participant is often emphasized in the literature and is interpreted as a significant prerequisite across the various procedures that have been used to study Inc-BiN (Longano & Greer., 2014; Greer at al., 2007; Greer & Du, 2015). Ensuring visual contact from a participant is relevant in the context of dyadic interactions when the experimenter is actively engaging with the child by pointing and/or looking at objects while saying their names. On the other hand, in the natural environment children may simply learn the names of objects by observation alone without necessarily being involved in a dyadic interaction (see, for example, Akhtar [2005] for an analysis on learning names through overhearing). Consider, for example, a situation in which two or more adults are interacting with each other while the child is present but not directly part of the interaction (e.g., when one adult asks a second to pass them “the corkscrew”). If the child observes this interaction, it is possible that they may learn the name of the object (i.e., corkscrew) even though neither of the adults were attempting to engage the child in joint attention towards the named object. Of course, it is likely that the child needs to attend to the interaction between the adults to learn the name, but this type of name learning, in which joint attention is not explicitly required or established as part of a dyad interaction, seems to require systematic experimental analysis. Although there have been conceptual and empirical studies on observational learning in behavior analysis (e.g., Fryling et al., 2011; Rothstein & Gautreaux, 2007; Taylor & DeQuinzio, 2012), this analysis remains to be extended to the study of incidental naming.

In developmental psychology there is evidence that suggests that children, some as young as 18 months of age who are observing third-party interactions can learn novel words (Floor & Akhtar, 2009; Akhtar, 2005) and novel actions (Herold & Akhtar, 2008). These studies highlight the extent and scope of a child’s incidental learning of names through observation in the natural environment. Within behavior analysis, however, there have been no studies that directly tested incidental naming through overhearing. On balance, researchers have conducted studies in which target children watch a peer receive instruction on tact trials (i.e., a teacher presents a novel picture to a peer along with the instruction “What is this?,” and provides prompts if necessary and reinforcement for the peer’s correct responses) and are then probed for their own tact responses to the same stimuli (Rothstein & Gautreaux, 2007; see also Greer & Ross, 2008). But these situations typically involved the target child being instructed to attend to the teacher and the peer. It seems essential for the behavioral literature to systematically investigate how children learn names incidentally across a range of ecological situations including observing/overhearing other people interacting in their environment. For instance, we argued earlier that Crel cues such as pointing and saying “That is a [object name]” control listener and speaker responding over naturalistic experiences that simulate multiple exemplar training. What might be the behavioral history that facilitates correct responding in a child watching a naming experience that involves one adult passing a corkscrew to another upon request?

Second, a common variable across all naming studies, including those mentioned in the previous section, has involved presenting the object and its name simultaneously. As noted above, Sivaraman et al. (2021) argued that when a name and an object are presented simultaneously, learning that name may not require contextually controlled derived bidirectional relations between the object and its name. It must be admitted that this issue could be seen as rather technical and relevant to a particular theoretical perspective (i.e., relational frame theory). However, it remains the case that children likely learn the names of objects and events when the two stimuli (object and name) are not present simultaneously (e.g., during a drive in the countryside, a parent might say to their child, “That was a horse” when the animal is no longer in view). As such, it seems important to analyze these types of naming experiences where there is a delay between the presentation of the object and its name. In conducting research in this area it seems likely that the role of contextual cues will be important in “bridging the temporal gap” between the name and the object. In the example above, the phrase “That was a” could be critical in establishing the name for a stimulus that is no longer present in the child’s visual field. It is clear that much experimental work remains to be done to explore the role played by contextual cues in establishing successful naming in such contexts.

Third, multiple stimuli have typically been presented together during the naming experience and tests in studies of Inc-BiN (Kleinert-Ventresca et al., 2023; Pérez-González et al., 2014; Morgan et al., 2021), which may confound learning to name novel objects per se with a child’s ability to “remember” multiple names for multiple objects. Although remembering multiple names is clearly an important skill or ability for children to acquire, it may be useful to explore Inc-BiN using procedures that require learning only one name at a time. Once single-name learning is reasonably well-established then progressing to multiple-name/object learning may be appropriate. It is important to clarify at this point that we are not suggesting that researchers test children on their ability to learn only one novel name and use that as conclusive evidence for incidental naming. Testing across multiple novel exemplars is critical to identify whether incidental naming is truly present as a higher order operant, but we are suggesting that each test administration is carried out with one stimulus at a time and multiple such tests be administered rather than four or five different novel items being presented together during one naming test (see Luciano et al., 2007 or Sivaraman et al., 2021 for examples of naming tests with a single stimulus at a time).

In studying single name learning, it would also seem important to explore the role of the previous two variables (joint attention and nonsimultaneous presentations), including the role of contextual cues (e.g., to bridge temporal gaps) and how to facilitate Inc-BiN when a child is not directly involved in a dyad. As argued previously, RFT suggests that these types of variables may function as powerful contextual cues for naming itself. Therefore, it seems wise to vary these types of variables systematically.

## Future Research

In considering the three different variables listed above, which may be important in developing a more complete understanding of Inc-BiN, future studies may expand research in this area by systematically manipulating all the variables involved in a naming experience. In particular, examining the role of specific contextual cues presented during a naming experience and their impact in the context of dyad interactions seems to be critical. At the time of writing, only one published study (Sivaraman et al., 2021) has examined naming in which names and objects were presented nonsimultaneously with a brief delay between the name and object (Petursdottir et al., 2020, Experiments 1 and 2, presented the stimuli nonsimultaneously but without any delays). The former study employed typically developing toddler participants, and they each required MET to establish successful listener naming using the nonsimultaneous format. In this study, however, the potential role played by specific contextual cues for naming was not explored. Thus, future studies could analyze the impact of such cues with toddlers, older children, and even adults. That is, would younger and older children, and perhaps even adults, be influenced by the presence versus absence of particular cues, such as pointing (at the named object) and using phrases such as “that was” (while pointing)? For example, will successful naming be reduced when these typical cues are absent? To the authors knowledge there is virtually no research that has focused on the role of contextual cues and the impact of such cues when they are manipulated systematically in an experimental context. Given the lack of research in this area, particularly in the context of the nonsimultaneous naming procedure, it seems important to explore the role of linguistic and paralinguistic cues presented during a naming experience on the emergence of incidental naming.

It would be presumed that it might seem likely that the nonsimultaneous presentation format, combined with the manipulation of cues, may have little impact on adults with more extensive verbal behavior histories. However, it does seem important to test this assumption, which at the very least will allow for comparisons with the naming abilities of children on similar tasks. Furthermore, identifying what may be critical controlling variables, in terms of specific contextual cues for naming, may have important implications for enhancing children’s vocabulary learning speeds. In addition, in accordance with RFT, appropriate forms of MET could be implemented in which contextual control by cues would be reinforced across exemplars. Tests could subsequently be conducted to determine if the contextual control generalized to novel exemplars in the absence of direct reinforcement, thus establishing Inc-Bin where it was previously absent. This type of research could thus assist in remediating any deficits in naming ability that may consequently improve emergent naming behaviors in the natural environment by establishing sensitivity to the relevant contextual cues for naming itself.

Another area of future research that seems critically important in the context of Inc-BiN is to explore how it emerges across a wider range of situations in the daily lives of young children. As noted previously, children’s verbal learning histories are not explicitly formed from dyad interactions, in which a caregiver actively engages with a child in teaching them a new name for a novel object. That is, children may learn the new names for things and events simply by observing interactions among other speakers within the verbal community (e.g., when one adult asks another adult to pass them “the corkscrew”). As far as the authors are aware, this type of “attentive-overhearing” incidental naming (i.e., the naming experience is not directed at the child and there are no specific instructions given to the child to attend to other individuals) has not been widely investigated within the behavior analytic literature. Previous studies have employed yoked-contingency procedures to establish incidental naming based on observing interactions between two individuals (e.g., Rothstein & Gautreaux, 2007), but these involve explicitly directing the child to attend to the interaction (e.g., as part of a board game). In this respect, learning to name novel stimuli based on an interaction between two adults, in which the child is not directly involved, could be a new explorative domain to consider. In such cases, two adults would be directly interacting in naming events with one another but are not directly interacting with the child, although a critical requisite would be the child observing the naming event occurring in the adult interaction. It is clear that this type of investigation would extend beyond the dyadic naming-experience interaction described in previous studies. However, once again, exploring the role of various contextual cues, and delays in a nonsimultaneous presentation format, and also initiating MET if required, could be explored in the context of attentive-overhearing incidental naming experiences. Analyzing the critical variables that allow children to learn the names of novel objects simply by observing the naming behaviors of adults (who are not engaging directly with a child) could help to further develop the generalized and flexible skill of incidental naming in young children.

## Conclusion

The current article has sought to present an overview on conceptual and empirical analyses within the behavior analytic naming literature. As described above the distinction between bidirectional naming and incidental naming has amassed a systematic classification of naming into six subtypes in recent years. The proposed taxonomy seems like a productive way forward to enhance the technical language of such naming studies. Although we believe, that going forward in incidental naming, additional actions from the different behavioral theoretical perspectives should be considered. In particular, it seems beneficial for VBDT and RFT researchers to draw from each perspective, collaborating in research to further advance the understanding of the variables at play during the development of incidental naming, a critically important verbal behavioral cusp. Exploring the potential role of contextual cues in a naming experience is an avenue that could improve precision in the conditions that generate the complex patterns of relational responding involved in incidental naming.

## Footnotes

1 Miguel (2016) also suggested another subtype of naming “intraverbal naming,” which refers to naming that involves relating two or more stimuli in some way. For present purposes, the current article will focus on bidirectional naming and its various subcomponents.

2 We have chosen to limit our description to the types of naming experiences reported in the literature and did not go into other details such as the types of trials used to test listener and speaker responses following the naming experience, or the training methods used to teach incidental naming. We feel that going into these details may serve to distract the reader from the main arguments presented in the current article. Besides, these details have recently been reported in a review by Sivaraman and Barnes-Holmes (2023).

## References

[CR1] Akhtar, N. (2005). The robustness of learning through overhearing. *Developmental Science,**8*(2), 199–209. 10.1111/j.1467-7687.2005.00406 15720377 10.1111/j.1467-7687.2005.00406.x

[CR2] Barnes-Holmes, D., & Barnes-Holmes, Y. (2000). Explaining complex behavior: Two perspectives on the concept of generalized operant classes. *The Psychological Record,**50*, 251–265.

[CR3] Barnes-Holmes, D., Barnes-Holmes, Y., & Cullinan, V. (2000). Relational frame theory and Skinner’s *Verbal Behavior*: A possible synthesis. *The Behavior Analyst,**23*(1), 69–84. 10.1007/BF03392000 22478339 10.1007/BF03392000 PMC2731367

[CR4] Barnes-Holmes, D., Finn, M., McEnteggart, C., & Barnes-Holmes, Y. (2018). Derived stimulus relations and their role in a behavior-analytic account of human language and cognition. *Perspectives on Behavior Science,**41*(1), 155–173. 10.1007/s40614-017-01247 32004360 10.1007/s40614-017-0124-7 PMC6701495

[CR5] Barnes-Holmes, D., & Harte, C. (2022). Relational frame theory 20 years on: The Odysseus voyage and beyond. *Journal of the Experimental Analysis of Behavior,**117*(2), 240–266. 35014700 10.1002/jeab.733

[CR6] Barnes-Holmes, D., O’Hora, D., Roche, B., Hayes, S. C., Bissett, R. T., & Lyddy, F. (2001). Understanding and verbal regulation. In S. C. Hayes, D. Barnes-Holmes, & B. Roche (Eds.), *Relational frame theory: A post-Skinnerian account of human language and cognition* (pp. 103–117). Plenum. 10.1016/s0065-2407(02)80063-5 11605362

[CR7] Cao, Y., & Greer, R. D. (2018). Mastery of echoics in Chinese establishes bidirectional naming in Chinese for preschoolers with naming in English. *Analysis of Verbal Behavior,**34*, 79–99. 31976216 10.1007/s40616-018-0106-1 PMC6702488

[CR8] Chomsky, N. (1959). Review of Skinner’s *Verbal Behavior*. *Language,**35*, 26–58.

[CR9] Cooper, J. O., Heron, T. E., & Heward, W. L. (2020). *Applied behavior analysis*. Pearson UK.

[CR10] Fiorile, C. A., & Greer, R. D. (2007). The induction of naming in children with no prior tact responses as a function of multiple exemplar histories of instruction. *Analysis of Verbal Behavior,**23*(1), 71–87. 10.1007/BF03393048 22477382 10.1007/BF03393048 PMC2774606

[CR11] Floor, P., & Akhtar, N. (2009). Can eighteen-month-olds learn words by listening in on conversations? *Infancy,**9*, 327–339. 10.1207/s15327078in0903_4 33412677

[CR12] Fryling, M. J., Johnston, C., & Hayes, L. J. (2011). Understanding observational learning: an interbehavioral approach. *Analysis of verbal behavior,**27*(1), 191–203. 10.1007/BF03393102 22532764 10.1007/BF03393102 PMC3139552

[CR13] Ganger, J., & Brent, M. R. (2004). Reexamining the vocabulary spurt. *Developmental Psychology,**40*(4), 621–632. 10.1037/0012-1649.40.4.621 15238048 10.1037/0012-1649.40.4.621

[CR14] Gibbs, A. R., Tullis, C. A., Conine, D. E., & Fulton, A. A. (2023, March 17). A systematic review of derived relational responding beyond coordination in individuals with autism and intellectual and developmental disabilities. *Journal of Developmental & Physical Disabilities*, 1–36. 10.1007/s10882-023-09901-z PMC10020770 37361456

[CR15] Gilic, L., & Greer, R. D. (2011). Establishing naming in typically developing two-year-old children as a function of multiple exemplar speaker and listener experiences. *Analysis of Verbal Behavior,**27*, 157–177. 10.1007/BF03393099 22532761 10.1007/BF03393099 PMC3139556

[CR16] Greer, R. D., & Du, L. (2015). Experience and the onset of the capability to learn names incidentally by exclusion. *The Psychological Record,**65*(2), 355–373. 10.1007/s40732-014-0111-2

[CR17] Greer, R. D., & Longano, J. (2010). A rose by naming: how we may learn how to do it. *The Analysis of Verbal Behavior,**26*(1), 73–106. 10.1007/BF03393085 22477465 10.1007/BF03393085 PMC2900947

[CR18] Greer. R. D., Pohl, P. Du, L., & Moschella, J. L. (2017). The separate development of children’s listener and speaker behavior and the intercept as behavioral metamorphosis. *Journal of Behavioral & Brain Science*, *7*, 674–704. 10.4236/jbbs.2017.

[CR19] Greer R. D, & Ross D. E. (2008). *Verbal behavior analysis: Inducing and expanding complex communication in children with severe language delays*. Allyn & Bacon.

[CR20] Greer, R. D., & Speckman, J. (2009). The integration of speaker and listener responses: A theory of verbal development. *The Psychological Record,**59*, 449–488.

[CR21] Greer, R. D., Stolfi, L., Chavez-Brown, M., & Rivera-Valdes, C. (2005). The emergence of the listener to speaker component of naming in children as a function of multiple exemplar instruction. *Analysis of Verbal Behavior,**21*(1), 123–134. 10.1007/BF03393014 22477318 10.1007/BF03393014 PMC2774093

[CR22] Greer, R. D., Stolfi, L., & Pistoljevic, N. (2007). Emergence of Naming in preschoolers: A comparison of multiple and single exemplar instruction. *European Journal of Behavior Analysis,**8*, 119–131.

[CR23] Hawkins, E., Gautreaux, G., & Chiesa, M. (2018). Deconstructing common bidirectional naming: A proposed classification framework. *Analysis of Verbal Behavior,**34*(1–2), 44–61. 10.1007/s40616-018-0100-7 31976214 10.1007/s40616-018-0100-7 PMC6702485

[CR24] Hawkins, E., Kingdorf, S., Charnock, J., Szabo, M., & Gautreaux, G. (2009). Effects of multiple exemplar instruction on naming. *European Journal of Behavior Analysis,**10*(2), 265–273.

[CR25] Hayes, S. C., Barnes-Holmes, D., & Roche, B. (Eds.). (2001). *Relational frame theory: A post-Skinnerian account of human language and cognition.* Plenum Press. 10.1016/s0065-2407(02)80063-5 11605362

[CR26] Herold, K. H., & Akhtar, N. (2008). Imitative learning from a third-party interaction: Relations with self-recognition and perspective taking. *Journal of Experimental Child Psychology,**101*(2), 114–123. 18635193 10.1016/j.jecp.2008.05.004 PMC2577159

[CR27] Horne, P. J., Hughes, J. C., & Lowe, C. F. (2006). Naming and categorization in young children: IV: Listener behavior training and transfer of function. *Journal of the Experimental Analysis of Behavior,**85*(2), 247–273. 10.1901/jeab.2006.125-04 16673828 10.1901/jeab.2006.125-04 PMC1472628

[CR28] Horne, P. J., & Lowe, C. F. (1996). On the origins of naming and other symbolic behavior. *Journal of the Experimental Analysis of Behavior,**65*(1), 185–241. 10.1901/jeab.1996.65-185 16812780 10.1901/jeab.1996.65-185 PMC1350072

[CR29] Hotchkiss, R. M., & Fienup, D. M. (2020). A parametric analysis of a protocol to induce bidirectional naming: Effects of protocol intensity. *The Psychological Record,**70*, 481–497.

[CR30] Iverson, J. M., & Goldin-Meadow, S. (2005). Gesture paves the way for language development. *Psychological Science,**16*(5), 367–371. 15869695 10.1111/j.0956-7976.2005.01542.x

[CR31] Kleinert-Ventresca, K., Greer, R. D., & Baldonado, L. (2023). More complex incidental bidirectional naming results from exposure alone. *Journal of the Experimental Analysis of Behavior,**119*(3), 461–475. 10.1002/jeab.847 10.1002/jeab.847 37186305

[CR32] Lamarre, J., & Holland, J. G. (1985). The functional independence of mands and tacts. *Journal of the Experimental Analysis of Behavior,**43*(1), 5–19. 10.1901/jeab.1985.43-5 16812407 10.1901/jeab.1985.43-5 PMC1348092

[CR33] Lodhi, S., & Greer, R. D. (1989). The speaker as listener. *Journal of the Experimental Analysis of Behavior,**51*(3), 353–359. 10.1901/jeab.1989.51-353 16812582 10.1901/jeab.1989.51-353 PMC1338927

[CR34] Longano, J. M., & Greer, R. D. (2014). Is the source of reinforcement for naming multiple conditioned reinforcers for observing responses? *Analysis of Verbal Behavior,**31*(1), 96–117. 10.1007/s40616-014-0022-y 27606200 10.1007/s40616-014-0022-y PMC4883541

[CR35] Longano, J. M., & Greer, R. D. (2015). Is the source of reinforcement for naming multiple conditioned reinforcers for observing responses? *Analysis of verbal behavior,**31*, 96–117. 27606200 10.1007/s40616-014-0022-y PMC4883541

[CR36] Luciano, C., Gómez Becerra, I., & Rodríguez Valverde, M. (2007). The role of multiple-exemplar training and naming in establishing derived equivalence in an infant. *Journal of the Experimental Analysis of Behavior,**87*(3), 349–365. 10.1901/jeab.2007.08-06 17575901 10.1901/jeab.2007.08-06 PMC1868588

[CR37] McLaughlin, S. F. (2010). Verbal behavior by B.F. Skinner: Contributions to analyzing early language learning. *Journal of Speech & Language Pathology—Applied Behavior Analysis, 5*(2), 114–131. 10.1037/h0100272

[CR38] McMurray, B. (2007). Defusing the childhood vocabulary explosion. *Science,**317*(5838), 631. 17673655 10.1126/science.1144073

[CR39] Miguel, C. F. (2016). Common and intraverbal bidirectional naming. *Analysis of Verbal Behavior,**32*(2), 125–138. 30800621 10.1007/s40616-016-0066-2 PMC6381345

[CR40] Miguel, C. F. (2018). Problem-solving, bidirectional naming, and the development of verbal repertoires. *Behavior Analysis: Research & Practice,**18*(4), 340–353. 10.1037/bar0000110

[CR41] Morford, M., & Goldin-Meadow, S. (1992). Comprehension and production of gesture in combination with speech in one-word speakers. *Journal of Child Language,**19*(3), 559–580. 1429948 10.1017/s0305000900011569

[CR42] Morgan, G. A., Greer, R. D., & Fienup, D. M. (2021). Descriptive analyses of relations among bidirectional naming, arbitrary, and nonarbitrary relations. *Psychological Record,**71*, 367–387. 10.1007/s40732-020-00408-z

[CR43] Morris, E. K., Smith, N. G., & Altus, D. E. (2005). B. F. Skinner's contributions to applied behavior analysis. *The Behavior Analyst*, *28*(2), 99–131. 10.1007/BF0339210 10.1007/BF03392108 PMC2755377 22478444

[CR44] Olaff, H. S., & Holth, P. (2020). The emergence of bidirectional naming through sequential operant instruction following the establishment of conditioned social reinforcers. *Analysis of Verbal Behavior,**36*(1), 21–48. 10.1007/s40616-019-00122-0 32699737 10.1007/s40616-019-00122-0 PMC7343676

[CR45] Olaff, H. S., Ona, H. N., & Holth, P. (2017). Establishment of naming in children with autism through multiple response-exemplar training. *Behavioral Development Bulletin,**22*(1), 67–85. 10.1037/bdb0000044

[CR46] Palmer, D. C. (2006). On Chomsky’s appraisal of Skinner’s *Verbal Behavior*: A half century of misunderstanding. *The Behavior Analyst,**29*, 253–267. 22478467 10.1007/BF03392134 PMC2223153

[CR47] Pérez-González, L. A., Cereijo-Blanco, N., & Carnerero, J. J. (2014). Emerging tacts and selections from previous learned skills: A comparison between two types of naming. *Analysis of Verbal Behavior,**30*(2), 184–192. 10.1007/s40616-014-0011-1 27274978 10.1007/s40616-014-0011-1 PMC4883521

[CR48] Petursdottir, A. I., Neaves, S. M., & Thomas, O. N. (2020). Emergent tact control following stimulus pairing: Comparison of procedural variations. *Analysis of Verbal Behavior,**36*(2), 193–214. 10.1007/s40616-020-00132-3 33381380 10.1007/s40616-020-00132-3 PMC7736392

[CR49] Rothstein, M. B., & Gautreaux, G. G. (2007). The effects of a peer-yoked contingency on observational learning and the collateral emergence of naming. *Journal of Early & Intensive Behavior Intervention,**4*(2), 453.

[CR50] Sidman, M. (1994). *Equivalence relations and behavior: A research story.* Authors Cooperative.

[CR51] Sivaraman, M., Barnes-Holmes, D., Greer, R. D., Fienup, D. M., & Roeyers, H. (2023). Verbal behavior development theory and relational frame theory: Reflecting on similarities and differences. *Journal of the Experimental Analysis of Behavior,**119*(3), 539–553. 10.1002/jeab.836 36808741 10.1002/jeab.836

[CR52] Sivaraman, M., Barnes-Holmes, D., & Roeyers, H. (2021). Non-simultaneous stimulus presentations and their role in listener naming. *Journal of the Experimental Analysis of Behavior,**116*(3), 300–313. 10.1002/jeab.715 34542178 10.1002/jeab.715

[CR53] Skinner, B. F. (1957). *Verbal behavior*. Copley Publishing Group & the B. F.

[CR54] Skinner, B. F. (1978). *Reflections on behaviorism and society*. Prentice Hall.

[CR55] Stewart, I. (2018). Derived relational responding and relational frame theory: A fruitful behavior analytic paradigm for the investigation of human language. *Behavior Analysis: Research & Practice,**18*(4), 398–415. 10.1037/bar0000129

[CR56] Sundberg, M. L., & Michael, J. (2001). The benefits of Skinner’s analysis of verbal behavior for children with autism. *Behavior Modification,**25*(5), 698–724. 11573336 10.1177/0145445501255003

[CR57] Taylor, B. A., & DeQuinzio, J. A. (2012). Observational learning and children with autism. *Behavior Modification,**36*(3), 341–360. 10.1177/0145445512443981 22569578 10.1177/0145445512443981

[CR58] Törneke, N. (2010). *Learning RFT: An introduction to relational frame theory and its clinical application.* Context Press/New Harbinger.

[CR59] Woodward, A., Markman, E. M., & Fitzsimmons, C. M. (1994). Rapid word learning in 13- and 18-month-olds. *Developmental Psychology,**30*, 553–566.

[CR60] Yoon, J. S., Greer, R. D., Virk, M., & Fienup, D. M. (2023). The establishment of incidental bidirectional naming through multiple exemplar instruction: A systematic replication. *Analysis of Verbal Behavior,**39*, 86–98. 37397134 10.1007/s40616-023-00181-4 PMC10313582

